# Effect of coated-benzoic acid on growth performance, immunity, and intestinal functions in weaned pigs challenged by enterotoxigenic *Escherichia coli*

**DOI:** 10.3389/fvets.2024.1430696

**Published:** 2024-09-16

**Authors:** Jiawen Qi, Bing Yu, Youjun Hu, Yuheng Luo, Ping Zheng, Xiangbing Mao, Jie Yu, Xiaonan Zhao, Taiqian He, Hui Yan, Aimin Wu, Jun He

**Affiliations:** ^1^Institute of Animal Nutrition, Sichuan Agricultural University, Chengdu, China; ^2^Key Laboratory of Animal Disease-Resistant Nutrition, Chengdu, China; ^3^Nuacid Nutrition Co., Ltd, Qingyuan, China

**Keywords:** immunity, intestinal epithelium, coated benzoic acid, weaned pigs, enterotoxigenic *Escherichia coli*

## Abstract

**Introduction:**

Benzoic acid (BA) could be added to the diets of weaned pigs to prevent diarrhea due to its antibacterial function. However, BA may be absorbed or decomposed before it can reach the hindgut. This study was conducted to explore the effect of a novel coated benzoic acid (CBA) on growth performance, immunity, and intestinal barrier functions in weaned pigs upon enterotoxigenic *Escherichia coli* (ETEC) challenge.

**Methods:**

In a 21d experiment, 32 piglets were randomly assigned to 4 treatments: (1) a basal diet (CON), (2) CON added with CBA at 3  g/kg (CBA); (3) CON and challenged by ETEC (ECON); (4) CON added with CBA at 3 g/kg and challenged by ETEC (ECON). On d 22, all piglets were euthanised to obtain samples.

**Results:**

Dietary CBA supplementation elevated the average daily gain (ADG) of the ETEC-challenged pigs (*p* < 0.05). CBA also improved the digestibility of dry matter, gross energy, and ash (*p* < 0.05). Moreover, CBA elevated the ratio of blood basophil and the serum concentration of total cholesterol of the ETEC challenged pigs (*p* < 0.05). Importantly, CBA increased the serum concentrations of immunoglobulin A (IgA), IgG, and IgM (*p* < 0.05). CBA not only decreased the crypt depth but also increased the ratio of villus height to crypt depth (V:C) in the jejunum and ileum (*p* < 0.05). Moreover, CBA increased the activities of jejunal and ileal sucrase, and the activities of duodenal and ileal maltase (*p* < 0.05). Importantly, CBA elevated the expression levels of critical functional genes such as the claudin-1, occluding, glucose transporter-2 (GLUT2), and sodium/glucose cotransporter-1 (SGLT-1) in the jejunal epithelium upon ETEC challenge (*p* < 0.05). Additionally, CBA increased the abundances of total bacteria and *Bacillus*, and increased the concentrations of volatile fatty acids (acetic acid, propanoic acid, and butyric acid) in cecum (*p* < 0.05).

**Discussion:**

These results suggested a beneficial role for CBA in alleviating intestinal injury in weaned pigs following ETEC challenge. Such effects may be tightly associated with elevated immunity and improved intestinal epithelium functions and microbiota.

## 1 Introduction

Weaning is a critical challenge for piglets as it causes abrupt changes in their gastrointestinal tract, disrupting their gut microbiota and mucosal immune function. This disruption can lead to a reduction in feed intake and growth and the development of post-weaning diarrhea (PWD) (1). Enterotoxigenic *Escherichia coli* (ETEC), a highly proliferating pathogenic bacterial strain, is one of the major bacterial causes of PWD (2). The main virulence attributes of ETEC are adhesins and enterotoxins (3, 4). It has been shown that ETEC can adhere to intestinal epithelial cells and cause intestinal injury (5). In the last decades, various antibiotics have been supplemented in the diet of weaned pigs to relieve diarrhea and intestinal injury associated with ETEC infection (6). However, numerous countries and regions have banned the use of antibiotics in the feed of swine, since it has been suggested that the continuous use of antibiotics may result development of resistant pathogen strains and drug residues in animal products (7). Therefore, there is an urgent need for alternatives to traditionally used antibiotics, and various alternatives such as acidifiers, probiotics, prebiotics, and plant extracts have attracted considerable research interest worldwide (8).

As a potentially attractive alternative to conventional antibiotics, acidifiers have been widely utilized in farm production for a long time. Benzoic acid (BA) is an aromatic carboxylic acid that occurs naturally in plant and animal tissues and can also be produced by microorganisms (9), and possesses a wide range of biological activities. For industrial production, BA is produced via oxidation of toluene with air at high temperature and pressure with the use of heavy metal catalysts such as cobalt or manganese naphthenate (10). It has been shown that BA could improve growth performance and intestinal health, and exert antibacterial activity by decreasing pH levels of the stomach and gut digesta (11). However, there are some problems with the oral administration of various acidifiers. One of the biggest challenges is the acidic environment of the stomach, which can degrade free organic acids, including BA, before they arrive in the small intestine (12). Cold spray technology is a powerful powder consolidation process for coating development and has been widely utilized for manufacturing various drug-loaded pellets for oral administration (13). A mixture of medium and long-chain fatty acids is stable and has been widely utilized for coating various unstable substances, which may allow the acidifiers to exert their effects in the distal small intestine and hindgut (14). Although numerous studies on BA showed a beneficial effect on animal production (15–17), there are only a few reports of coated benzoic acid (CBA) on growth performance, immunity, and intestinal functions in weaned pigs upon bacterial infection (11). The objective of this study was to evaluate whether CBA supplementation could alleviate ETEC-induced intestinal inflammation and epithelial damage in weaned pigs. This study may help us further understand the mechanism through which CBA improves pig performance and provide the scientific basis for the beneficial effects of CBA.

## 2 Materials and methods

### 2.1 Animal care and use statement

The experimental procedures in the present investigation underwent evaluation and authorization by the Animal Experimental Committee of Sichuan Agricultural University (authorisation number SICAU-2022-014). All experiment procedures were conducted following the guidelines for the Care and Use of Laboratory Animals.

### 2.2 Bacterial strains and culture

The pathogenic ETEC strain was purchased from the China veterinary culture collection center (CVCC, Beijing, China). The bacterial inoculum used was prepared from *E. coli* K88+ strain (serotype O149:K91, K88ac). The freeze-dried strain was cultured in Luria–Bertani (LB) broth culture medium overnight with shaking at 250 × g at 37°C. After overnight incubation, ETEC K88+ was inoculated in fresh LB medium at 1:100 and cultured until med-logarithmic growth stage was reached, pelleted by centrifugation and the pellet was then sequentially washed three times in phosphate buffered saline (PBS) and resuspended to a concentration of 10^6^ CFU/mL bacteria (18).

### 2.3 Experimental design and diet

Thirty-two healthy barrows (Duroc × Landrace × Yorkshire) weaned on their 21st day and with an average body weight (BW) of 7.84 ± 0.14 kg were used in the experiment, which purchased from the farm of Sichuan Vanguard Food Co. The pigs were randomly allocated into a 2 × 2 factorial arrangement to four treatments (*n* = 8) composed of CON (the basal diet), CBA (CON added with coated benzoic acid at 3 g/kg), ECON (CON and challenged by ETEC), ECBA (CON added with coated benzoic acid at 3 g/kg and challenged by ETEC). CBA was kindly provided by Guangzhou Nuacid Co., Ltd. (Guangzhou, China). BA is safe for weaned piglets at 3 g/kg complete feed (19). The experimental period lasted 21 days. Pigs were stratified into individual 1.5 × 0.7 m^2^ metabolism cages, which were located in a controlled room, according to temperature (27 ± 1°C) and relative humidity (65 ± 5%). According to treatment and live weight and offered their respective diets. The diets (Table 1) were formulated to meet the National Research Council recommended nutrient requirements for pigs (20). Water and feed were provided *ad libitum* to the piglets. The ETEC-challenged groups were orally treated with 200 mL Luria-Bertani (LB) medium containing 1 × 10^6^ CFU/mL of ETEC on d 19, and the non-challenged groups were infused with an equal volume of LB medium.

**Table 1:** Experiment basal diet composition and nutrient level.

Ingredients	%	Nutrient level	Contents
Corn	26.73	Digestible energy, calculated, MJ/kg	3.55
Extruded corn	25.45	Crude Protein, %	19.80
Soybean meal	9.50	Calcium, %	0.92
Extruded full-fat soybean	10.50	Available phosphorus, %	0.37
Fish meal	4.00	Lysine, %	1.41
Whey powder	7.00	Methionine, %	0.47
Soybean protein concentrate	8.00	Methionine + cysteine, %	0.75
Soybean oil	2.00	Threonine, %	0.79
Sucrose	4.00	Tryptophan, %	0.22
Limestone	0.90		
Dicalcium phosphate	0.50		
NaCl	0.30		
L -Lysine HCl, 78%	0.47		
DL-Methionine	0.15		
L -Threonine, 98.5%	0.13		
Tryptophan, 98%	0.03		
Chloride choline	0.10		
Vitamin premix ^1^	0.04		
Mineral premix ^2^	0.20		
Total	100		

^1^The vitamin premix provided the following per kg of diet: 6000 IU of VA, 400 IU of VD 3, 10 IU of VE, 2 mg of VK 3, 0.8 mg of VB1, 6.4 mg of VB 2, 2.4 mg of VB6, 12 ug of VB12, 14 mg of niacin, 10 mg of pantothenic acid, 0.2 mg of folic acid. ^2^The mineral premix provided the following per kg of diet: 120 mg Fe, 6 mg Cu, 100 mg Zn, 40 mg Mn, 0.3 mg I, 0.3 mg Se. The diet was formulated based on the recommendation of NRC2012.

### 2.4 Growth performance evaluation

Individual pig body weight (BW) was recorded at d 1, d 19, and d 22 after all pigs were food-deprived for 12 h. Feed consumption was recorded as the amount of feed offered daily minus the remaining quantity the next morning during the experiment. These values were used to calculate average daily gain (ADG), average daily feed intake (ADFI), and feed efficiency (F/G).

### 2.5 Sample collection

Experimental diets were sampled and stored at −20°C until analysis. Fresh fecal samples were collected from d 15 to d 18 during the trial, weighed, and 10% hydrochloric acid was added for fixation of excreta nitrogen. The four feeds and fecal samples were dried at 65°C until constant weight, after which they were ground to pass through a 1-mm screen and then stored at −20°C until analysis of key nutrient contents. Blood samples were collected on day 22 via jugular vein puncture into two 10-mL nonheparinized vacuum tubes and one 5-mL vacuum tube containing ethylenediaminetetraacetic acid and its salts. The 5-mL blood sample was used for routine blood analysis, and the other blood samples were placed on ice, taken to the laboratory and centrifuged at 3500 × g for 15 min at 4°C to recover serum, which was stored at −20°C until the serum indexes analysis. Following blood collection, all the pigs were killed by intravenous injection with sodium pentobarbital (200 mg/kg BW). Approximately 4 cm segments from the middle of the duodenum, jejunum and ileum were quickly isolated, washed with cold PBS, and then fixed in 4% paraformaldehyde solution for morphological analysis. The remaining segments of the duodenum, jejunum, and ileum were opened longitudinally, washed with ice-cold PBS, and gently scraped with a sterile glass microscope slide at 4°C to obtain mucosa samples. The mucosa samples were immediately snap-frozen in liquid nitrogen and stored at −80°C until further analysis of related enzyme activities and real-time polymerase chain reaction (RT-PCR). Additionally, the intestinal contents from the colon and cecum were collected and stored at −80°C for further analysis.

### 2.6 Apparent total tract nutrient digestibility analysis

Feed and fecal samples were used for the apparent total tract digestibility (ATTD) analysis using acid insoluble ash (AIA) as an endogenous indicator (GB/T 23742–2009). The dry matter (930.15; AOAC), crude protein (930.15; AOAC), crude fat (920.39; AOAC) and crude ash (942.05, AOAC) contents were determined according to official methods of analysis of AOAC international (21). The gross energy concentration was determined using an automatic oxygen bomb calorimetry (parr6400-1101-22141). The ATTD of the nutrients was calculated as (100 – A1/A2 × F2/F1 × 100) (22), A1: digesta nutrient; A2: digesta AIA; F1: diet AIA; F2: digesta AIA.

### 2.7 Measurement of the hematological parameters

The routine blood test was implemented using an automatic biochemistry analyzer approximately 3 h post slaughter. The following hematological parameters were estimated: red blood cell count (RBC), hemoglobin concentration (HBC), packed cell volume (PCV), white blood cell count (WBC), neutrophil count (Neu), neutrophil count percentage, monocyte count (Mono), monocyte count percentage, lymphocyte count (Lym), lymphocyte count percentage, eosinophil count (Eos), eosinophil count percentage, basophil count (Baso), basophil count percentage, red blood cell counts (RBC), hematocrit (HCT), hemoglobin (HGB), mean corpuscular hemoglobin (MCH), mean corpuscular hemoglobin concentration (MCHC), mean corpuscular volume (MCV), coefficient of variation in red blood cell volume distribution width (RDW-CV), standard deviation of red blood cell volume distribution width (RDW-SD), platelet count (PLT).

### 2.8 Serum biochemistry and immunoglobulin detection

Total protein (TP), albumin (ALB), alkaline phosphatase (AKP), glutamic oxaloacetic transaminase (GOT), glutamic pyruvic transaminase (GPT), total cholesterol (TC), glucose (GLU), triglyceride (TG), and urea (UREA) were detected by the automatic biochemical analyser (Olympus, Shanghai, China). Total serum concentrations of immunoglobulin A (IgA, MM-0905O1), immunoglobulin M (IgM, MM-0402O1), and immunoglobulin G (IgG, MM-0403O1) were determined with commercial Enzyme-Linked Immunosorbent Assay (ELISA) kits (Shanghai Meimian Biotechnology Co., Ltd., Shanghai, China) according to the manufacturer’s recommendations. The standards provided in the kits were used to generate standard curves for quantification.

### 2.9 Histomorphology measurements of intestinal segments

The intestinal segments fixed with 4% paraformaldehyde were dehydrated through a graded series of ethanol and then embedded in paraffin (23). Cross-sections of each sample were prepared, stained with Hematoxylin and eosin (H&E), and then sealed with neutral resin. Ultrathin sections of the duodenal, jejunal and ileal samples were examined for crypt depth (CD) and villus height (VH) with an image processing and analysis system (Image-Pro Plus 6.0), and their ratio V/C was calculated (24). At least 20 intact, well-oriented villus heights and corresponding crypt depths were obtained per section for measurement.

### 2.10 Enzyme activity

Mucosal tissues from the duodenal, jejunal and ileal samples were homogenized in ice-cold saline using a tissue homogenizer, made into 10% homogenate, then all samples were centrifuged at 3500 × g at 4°C for 10 min. Commercially available colourimetric diagnostic kits (Nanjing Jiancheng Bioengineering Institute, Nanjing, China) were used to measure the activities of intestinal alkaline phosphatase (AKP). ELISA kits (Shanghai Enzyme-linked Biotechnology Co., Ltd., Shanghai, China) were used for the detection of lactase (Porcine Lactase ELISA Kit ml712060), sucrase (Porcine Sucrase ELISA Kit ml712026), and maltase (Porcine Maltase ELISA Kit ml712030). All procedures were carried out following the manufacturer’s instructions. One unit of enzyme activity was defined as the hydrolysis of 1 mol of substrate in 1 min at 37°C and a pH of 6.0. A spectrophotometer (UV–VIS Spectrophotometer, Leng Guang SFZ1606017568, Shanghai, China) at a wavelength of 450 nm was used to determine the absorbance of each reaction.

### 2.11 Hindgut microbiological analysis

The total genomic DNA for quantitative PCR was isolated from approximately 0.2 g cecal and colonic digesta using the commercially available stool DNA extraction kit, according to the manufacturer’s instructions (Omega Bio-Tek, Doraville, CA, USA), which was performed by conventional PCR on the CFX96 Real-Time PCR Detection system (Bio-Rad Laboratories, Hercules, CA, USA). Total bacteria PCRs were carried out in triplicate using 10 μL reactions with 5 μL SYBR Premix Ex Taq (2× concentrated), 0.4 μL of forward and reverse primers respective (100 nM), 1 μL DNA, 0.2 μL 50 × ROX Reference Dye*3 and 3 μL of RNase-Free ddH2O. The amplification program consisted of d 95°C for 25 s; followed by 40 cycles of 95°C for 5 s and 64.5°C for 25 s; and then a final melting curve for SYBR Green tests. *Lactobacillus*, *E. coli*, *Bacillus* and *Bifidobacterium* were detected by the SuperReal PreMix (Probe) kit (Tiangen Biotech Co., Ltd., Beijing, China). Supplementary Table S1 shows the primers and fluorescent oligonucleotide probes. Each reaction was run with three repeats in a volume of 20 μL with 10 μL 2 × Super Real PreMix (Probe), 0.6 μL of forward and reverse primers (100 nM) respective, 0.4 μL probe (100 nM), 2 μL DNA and 6.4 μL of RNase-Free ddH_2_O. All reaction protocol was composed of one cycle of pre-denaturation at 95°C for 15 min; 49 cycles of denaturation at 95°C for 3 s; annealing and extension at 53°C for 25 s. The Cycle threshold (Ct) values and baseline settings were determined by automatic analysis settings, and the copy numbers of the target group for each reaction were calculated from the standard curves, which were generated by constructing The Cycle threshold (Ct) values and baseline settings were determined by automatic analysis settings, and the copy numbers of the target group for each reaction were calculated from the standard curves, which were generated by constructing standard plasmids by a 10-fold serial dilution of plasmid DNA (1 × 10^1^ to 1 × 10^9^ copies/μL).

### 2.12 Analysis of volatile fatty acids in hindgut digesta

The Volatile fatty acids (VFA; acetic acid, propanoic acid, butyric acid) concentrations in the cecum and colon were analyzed by a gas chromatograph system (VARIAN CP-3800, Varian, Palo Alto, CA, USA; capillary column 30 m × 0.32 mm × 0.25 μm film thickness) as per previous study (25). The supernatant (1 μL) was analyzed using the gas chromatograph. The polyethene glycol column was operated with highly purified N_2_ as the carrier gas at 1 mL/min.

### 2.13 Isolation and reverse transcription of RNA and qPCR

Approximately 0.1 g of each frozen sample isolated from duodenum, jejunum, and ileum were rapidly homogenized in 1 mL of RNAiso Plus (Takara Biotechnology Co., Ltd., Dalian, China). RNA was extracted from the homogenized samples according to the manufacturer’s instructions. RNA concentration and purity were assessed using a spectrophotometer (NanoDrop 2000, Thermo Fisher Scientific, Inc., Waltham, MA, USA). Samples that had a 260/280 ratio of 1.8 to 2.0 were deemed appropriate. Subsequently, around 1 μg of total RNA from each duodenal, jejunal, and ileal sample was used for cDNA synthesis using the protocol of PrimeScript™ RT reagent kit with gDNA Eraser (Takara Biotechnology Co., Ltd., Dalian, China). This process was as follows: I: 37°C for 15 min, II: 85°C for 5 s. The expression level of the target gene in the intestinal mucosa was determined by qPCR, the oligonucleotide primers sequences used in qPCR are presented in Supplementary Table S1, qPCR was performed with the SYBR^®^ Green PCR I PCR reagents (Takara Bio Inc., Dalian, China) using a CFX96 Real-Time PCR Detection System (BioRad Laboratories, Hercules, CA, USA). All cDNA samples were run in triplicate. The reaction mixture (10 μL) was composed of 5 μL SYBR Premix Ex Taq II (Tli RNaseH Plus), 0.4 μL forward primer and reverse primer, 1 μL cDNA, 0.2 μL 50 × ROX Reference Dye*3 and 3 μL RNase-Free ddH2O. The procedure used in quantitative real-time qPCR was as follows: 95°C for 25 s, followed by 40 cycles: at 95°C for 5 s and 64.5°C for 25 s. After each real-time quantitative PCR assay, a melt curve analysis was included to confirm that only one amplicon was being generated. The mRNA relative expression level of target genes was standardized by the housekeeping gene β-actin, and calculated based on the 2^–∆∆Ct^ method (26).

### 2.14 Statistical analysis

Before the ETEC challenge the data was analyzed by one-way ANOVA. After the challenge, the data was analyzed by two-way ANOVA with the General Linear Model (GLM) procedure of SPSS as a 2 (CBA) × 2 (ETEC) factorial design. A *p* < 0.05 was regarded as significant and a *p*-value from 0.05 to 0.1 was regarded as a significant trend. Normality and variance homogeneity assumptions were confirmed using Shapiro–Wilk’s and Levene’s tests, respectively. Duncan’s multiple range test was used based on the analysis of ANOVA, which showed a significant difference. All data were analyzed by SPSS 27.0 (IBM, Chicago, IL, USA) and GraphPad (version 9) software (GraphPad Software Inc., CA, USA). Results are expressed as means with their standard errors.

## 3 Results

### 3.1 Effect of CBA on growth performance and nutrient digestibility in weaned pigs after ETEC challenge

Supplementation of CBA tended to a 21.89% ADG increase in weaned piglets before the ETEC challenge (*p* < 0.1; Table 2). The ETEC challenge showed a 54.59% ADG decrease in the piglets, but this effect was prevented by the CBA treatment (*p* < 0.05). CBA increased the digestibilities of DM (2.59%), CP (4.20%), Ash (10.34%), and GE (2.40%) in weaned piglets before the ETEC challenge (*p* < 0.05; Table 3).

**Table 2:** Effect of CBA supplementation on growth performance in weaned pigs before/after ETEC challenge.

ITEM	Treatments				SEM	*p*-value		
	CON	CBA	ECON	ECBA		CBA	ETEC	Interaction
1-19d								
Initial BW	7.99	7.78	7.66	7.85	0.07	0.373	–	–
ADFI, g/d	411.33	424.88	319.70-	411.11	17.22	0.13	–	–
ADG, g/d	245.14	286.46	192.86	251.67	12.78	0.08	–	–
F: G	1.74	1.51	1.70	1.66	0.05	0.39	–	–
19d BW	12.40^ab^	12.93^a^	11.13^b^	12.38^ab^	0.24	0.05	–	–
19-21d								
Final BW	13.03^a^	13.73^a^	11.41^b^	13.14^a^	0.27	0.014	0.025	0.279
ADFI, g/d	353.09	398.86	315.71	380.76	16.70	0.11	0.41	0.77
ADG, g/d	209.72^a^	264.58^a^	95.24^b^	252.50^a^	21.60	0.01	0.11	0.19
F: G	1.72	1.53	−1.04	1.56	0.43	0.14	0.10	0.09

BW, body weight; ADFI, average daily feed intake; ADG, average daily gain; F: G, feed: gain ratio.

Mean and total SEM are listed in Separate columns, 1-19d (*n* = 8); 19-21d (*n* = 8).

a, b, c mean values within a row with unlike superscript letters were significantly different, *p* < 0.05.

CON, pigs were fed with a basal diet; CBA, pigs were fed with a CBA containing diet, 3 g/kg; ECON, pigs were fed with a basal diet and challenged by ETEC; ECBA, pigs were fed with a CBA containing diet and challenged by ETEC.

**Table 3:** Effect of CBA supplementation on nutrient digestibility in weaned pigs before ETEC challenge.

ITEM	Treatments				SEM	*p*-value
	CON	CBA	ECON	ECBA		
DM, %	88.18^b^	90.46^a^	–	–	0.43	0.02
CP, %	83.83^b^	87.35^a^	–	–	0.90	0.20
EE, %	83.03	81.84	–	–	0.96	0.39
Ash, %	65.09^b^	71.82^a^	–	–	1.26	0.02
GE, %	88.32^b^	90.44^a^	–	–	0.47	0.03

DM, dry matter; CP, crude protein; EE, ether extract; GE, gross energy.

Mean and total SEM are list in Separate columns, *n* = 16.

a, b, c mean values within a row with unlike superscript letters were significantly different, *p* < 0.05.

CON, pigs were fed with a basal diet; CBA, pigs were fed with a CBA containing diet, 3 g/kg; ECON, pigs were fed with a basal diet and challenged by ETEC; ECBA, pigs were fed with a CBA containing diet and challenged by ETEC.

### 3.2 Effect of CBA on blood and serum parameters in weaned pigs after ETEC challenge

A trend toward an interaction effect of CBA and ETEC was observed that the increasing of WBC count upon ETEC challenge was prevented by the CBA treatment (*p* < 0.1; Table 4). CBA supplementation showed a 64.29% percentage of basophils increase in the ECBA group compared to the ECON group (*p* < 0.05).

**Table 4:** Effect of CBA supplementation on hematological parameters in weaned pigs after ETEC challenge.

ITEM	Treatments				SEM	*p*-value		
	CON	CBA	ECON	ECBA		CBA	ETEC	Interaction
WBC, 10^9^/L	19.77^a^	21.83^a^	18.07^ab^	14.19^b^	0.92	0.58	0.01	0.08
Neu, % of WBC	20.94	25.33	30.28	23.91	2.12	0.82	0.36	0.22
Lym, % of WBC	72.20	70.04	64.14	70.24	2.25	0.67	0.40	0.38
Mono, % of WBC	5.49	3.35	4.19	4.58	0.43	0.32	0.97	0.15
Eos, % of WBC	1.05	0.96	1.13	0.81	0.12	0.43	0.88	0.65
Baso, % of WBC	0.33^b^	0.33^b^	0.28^b^	0.46^a^	0.02	0.03	0.29	0.03
Neu, 10^9^/L	4.04^a^	5.20^a^	5.59^a^	3.22^ab^	0.42	0.46	0.80	0.04
Lym, 10^9^/L	14.43^ab^	15.67^a^	11.47^ab^	10.16^ab^	0.93	0.98	0.02	0.48
Mono, 10^9^/L	1.02	0.69	0.75	0.65	0.07	0.15	0.31	0.47
Eos, 10^9^/L	0.22	0.20	0.21	0.10	0.02	0.20	0.24	0.34
Baso, 10^9^/L	0.07	0.07	0.05	0.07	0.01	0.47	0.30	0.76
RBC, 10^12^/L	6.98^ab^	7.57^a^	6.88^b^	7.14^ab^	0.11	0.06	0.23	0.46
HGB, g/L	108.88	115.75	109.75	112.50	1.40	0.09	0.67	0.46
HCT, %	40.96^ab^	44.86^a^	40.16^b^	42.61^ab^	0.72	0.03	0.27	0.60
MCV, fL	58.63	59.24	58.64	59.70	0.52	0.44	0.83	0.84
RDW-SD, fL	43.06	41.46	38.71	41.28	0.97	0.81	0.26	0.30
RDW-CV, fL	23.24	23.39	21.33	22.16	0.48	0.61	0.12	0.72
MCH, pg	15.60	15.30	16.05	15.83	0.16	0.41	0.13	0.91
MCHC, g/L	266.25^ab^	259.00^b^	273.38^a^	264.75^ab^	2.16	0.06	0.13	0.87
PLT, 10^9^/L	425.25	407.13	393.63	434.25	17.78	0.76	0.95	0.43

WBC, White blood cell; Neu, Neutrophil; Lym, Lymphocyte; Mono, Monocyte; Eos, Eosinophil; Baso, Basophil; RBC, Red blood cell; HGB, Hemoglobin; HCT, Hematocrit; MCV, Mean corpuscular volume; RDW-SD, standard deviation of red blood cell volume distribution width; RDW-CV, coefficient of variation in red blood cell volume distribution width; MCH, Mean corpuscular hemoglobin; MCHC, Mean corpuscular hemoglobin concentration; PLT, blood platelet count.

Mean and total SEM are list in Separate columns, *n* = 8.

a, b, c mean values within a row with unlike superscript letters were significantly different, *p* < 0.05.

CON, pigs were fed with a basal diet; CBA, pigs were fed with a CBA containing diet, 3 g/kg; ECON, pigs were fed with a basal diet and challenged by ETEC; ECBA, pigs were fed with a CBA containing diet and challenged by ETEC.

CBA supplementation tended to increase serum albumin in both non-challenged (7.08%) and ETEC-challenged pigs (7.59%) (*p* < 0.1; Table 5). CBA supplementation increased serum total cholesterol (26.83%) and decreased serum urea (48.60%) in non-challenged pigs (*p* < 0.05). CBA supplementation significantly reduced serum urea levels (48.60%) in piglets under non-challenged conditions (*p* < 0.05) and also tended to reduce serum urea levels under ETEC-challenged conditions (*p* < 0.1).

**Table 5:** Effect of CBA supplementation on serum biochemistry in weaned pigs after ETEC challenge.

ITEM	Treatments				SEM	*p*-value		
	CON	CBA	ECON	ECBA		CBA	ETEC	Interaction
TP, g/L	40.98	42.30	40.31	40.65	0.83	0.64	0.51	0.78
ALB, g/L	23.43^ab^	25.09^a^	21.35^b^	22.97^ab^	0.58	0.16	0.07	0.99
AKP, U/L	141.38	163.75	158.25	134.88	6.74	0.97	0.66	0.10
GOT, U/L	28.63	35.40	32.44	32.55	2.32	0.14	0.25	0.40
GPT, U/L	39.20	41.33	35.43	33.35	1.86	1.00	0.13	0.58
TC, mmol/L	1.64^b^	2.08^a^	1.78^a^	2.01^a^	0.08	0.03	0.78	0.48
GLU, mmol/L	4.82	5.09	4.57	4.58	0.12	0.57	0.14	0.60
TG, μmol/L	0.49	0.45	0.35	0.40	0.03	0.93	0.10	0.43
UREA, mmol/L	3.56^a^	1.83^b^	3.39^a^	2.65^ab^	0.24	0.01	0.47	0.27

TP, total protein; ALB, albumin; AKP, alkline phosphatase; GOT, glutamic oxalacetic transaminase; GPT, glutamic pyruvic transaminase; TC, total cholesterol; GLU, glucose; TG, Triglyceride; UREA, urea.

Mean and total SEM are list in Separate columns, *n* = 8.

a, b, c mean values within a row with unlike superscript letters were significantly different, *P* < 0.05.

CON, pigs were fed with a basal diet; CBA, pigs were fed with a CBA containing diet, 3 g/kg; ECON, pigs were fed with a basal diet and challenged by ETEC; ECBA, pigs were fed with a CBA containing diet and challenged by ETEC.

**Figure 1:**
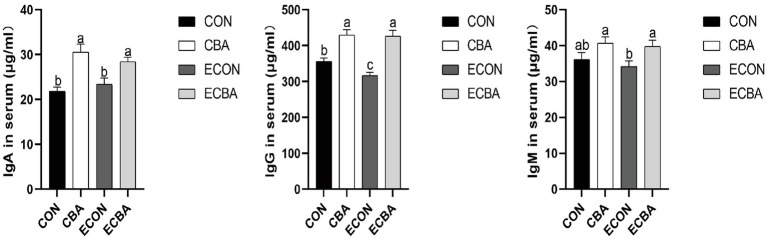
Effect of CBA supplementation on serum concentrations of immunoglobulins in weaned pigs after ETEC challenge. IgA, immunoglobulins A; IgG, immunoglobulins G; IgM, immunoglobulins M. a, b, c mean values within a row with unlike superscript letters were significantly different (*p* < 0.05). CON, pigs were fed with a basal diet; CBA, pigs were fed with a CBA containing diet, 3 g/kg; ECON, pigs were fed with a basal diet and challenged by ETEC; ECBA, pigs were fed with a CBA containing diet and challenged by ETEC.

CBA supplementation significantly increased the serum concentrations of IgG in both non-challenged and ETEC-challenged groups (*p* < 0.05; Figure 1). A trend toward an interaction effect of CBA and ETEC was observed that the decreasing of IgG upon ETEC challenge was prevented by the CBA treatment. CBA supplementation increased IgA and IgG concentrations under non-challenged conditions (*p* < 0.05) and increased the concentration of IgA and IgG, as tended to increase the concentration of IgM under ETEC-challenged conditions (*p* < 0.05).

### 3.3 Effect of CBA supplementation on intestinal morphology and mucosal enzyme activity in weaned pigs after ETEC challenge

**Figure 2:**
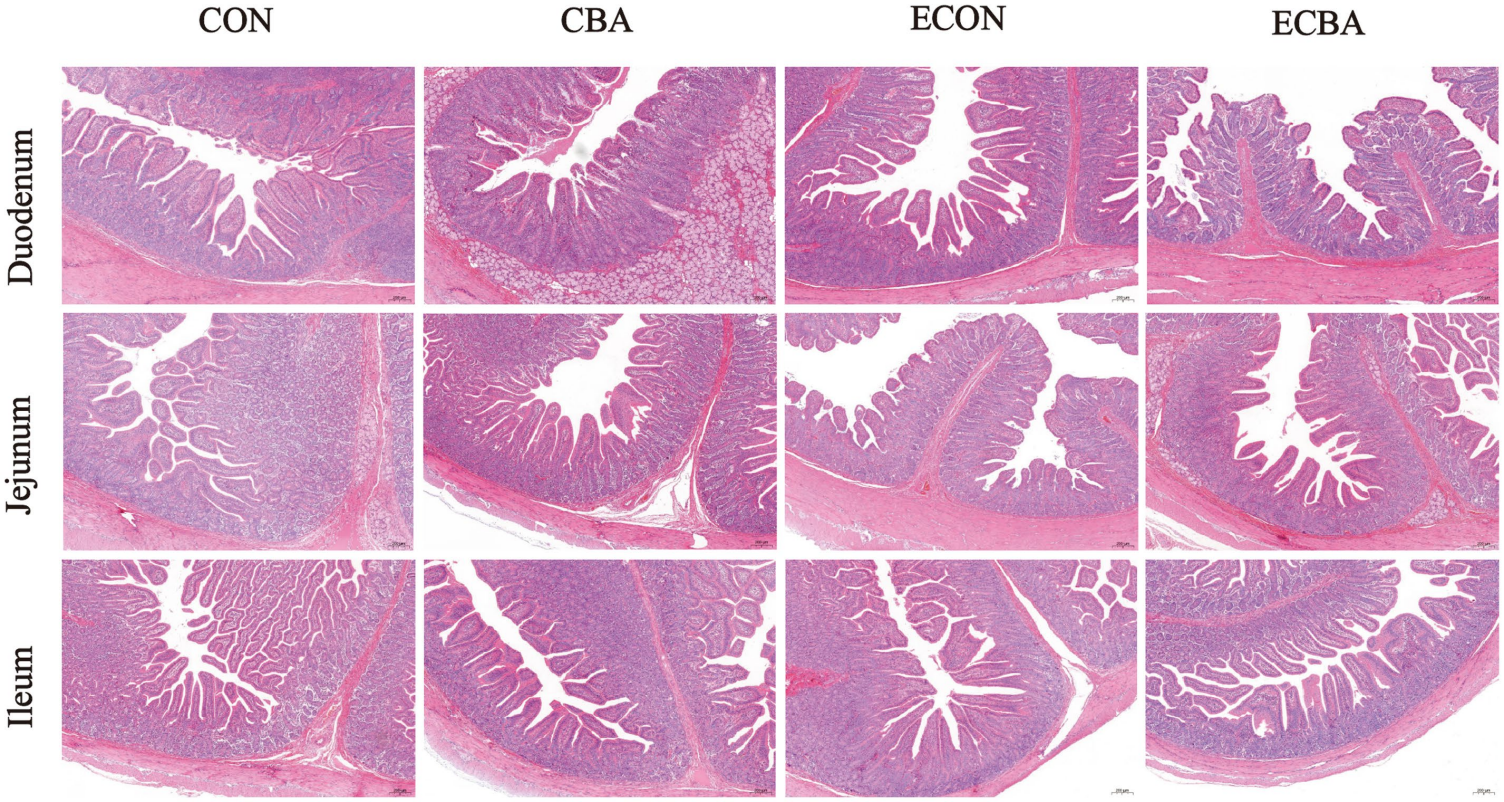
Effect of CBA supplementation on intestinal morphology in weaned pigs after ETEC challenge (H&E; ×40). CON, pigs were fed with a basal diet; CBA, pigs were fed with a CBA containing diet, 3 g/kg; ECON, pigs were fed with a basal diet and challenged by ETEC; ECBA, pigs were fed with a CBA containing diet and challenged by ETEC.

Two interaction effects of CBA and ETEC in duodenum were observed, when these interactions toward an effect were investigated with a post-hoc test, we observed that while ETEC challenge showed a 22.54% crypt depth increase and a 20.30% V:C decrease, these effects were prevented by the CBA treatment (*p* < 0.05; Table 6, Figure 2). CBA supplementation showed a 24.01% height of the ileum villi increase (*p* < 0.05), as well as the jejunal (10.98%) and ileal (32.53%) V:C of the non-challenged pigs (*p* < 0.05). ETEC challenge showed a 14.38% ileal villus height reduction and a 13.20% jejunal crypt depth increase in the ETEC-challenged pigs (*p* < 0.05). Nevertheless, pigs fed a diet supplemented with CBA had greater ileal villus height and jejunal crypt depth, then improved the jejunal (16.00%) and ileal (12.96%) V:C after the ETEC challenge (*p* < 0.05). ETEC challenge decreased the activities of maltase in the duodenum (42.03%) and sucrose in the jejunum (27.90%). However, CBA supplementation relieved the reduction of the activities upon ETEC challenge (*p* < 0.05; Table 7). CBA supplementation also enhanced the ileal activity of lactase (30.45%), sucrase (54.04%) and maltase (32.87%) upon ETEC challenge (*p* < 0.05), and of alkaline phosphatase (61.83%) without ETEC challenge (*p* < 0.05).

**Table 6:** Effect of CBA supplementation on intestinal morphology in weaned pigs after ETEC challenge.

ITEM	Treatments				SEM	*p*-value		
	CON	CBA	ECON	ECBA		CBA	ETEC	Interaction
Duodenum								
Villus height, *μ*m	422.85	401.95	411.03	421.58	5.54	0.66	0.74	0.19
Crypt depth, *μ*m	218.44^b^	201.67^b^	267.67^a^	205.82^b^	4.47	<0.01	<0.01	0.01
V: C	1.97^b^	2.14^ab^	1.57^c^	2.20^a^	0.03	<0.01	<0.01	<0.01
Jejunum								
Villus height, *μ*m	398.81^ab^	421.81^a^	382.34^b^	380.84^b^	4.51	0.327	<0.01	0.27
Crypt depth, *μ*m	252.69^ab^	239.29^ab^	264.85^a^	229.88^b^	3.75	<0.01	0.88	0.24
V: C	1.64 ± 0.08^bc^	1.82^a^	1.50^c^	1.74^ab^	0.03	<0.01	0.08	0.67
Ileum								
Villus height, *μ*m	354.01^c^	439.01^a^	340.78^c^	389.77^b^	5.82	<0.01	<0.01	0.10
Crypt depth, *μ*m	217.66	211.83	215.12	217.34	3.26	0.78	0.82	0.54
V: C	1.66^c^	2.20^a^	1.62^c^	1.83^b^	0.03	<0.01	<0.01	<0.01

V/C, Villus height: Crypt depth.

Mean and total SEM are list in Separate columns, *n* = 8.

a, b, c mean values within a row with unlike superscript letters were significantly different, P < 0.05.

CON, pigs were fed with a basal diet; CBA, pigs were fed with a CBA containing diet, 3 g/kg; ECON, pigs were fed with a basal diet and challenged by ETEC; ECBA, pigs were fed with a CBA containing diet and challenged by ETEC.

**Table 7:** Effect of CBA supplementation on mucosal enzyme activity in weaned pigs after ETEC challenge.

ITEM	Treatments				SEM	*p*-value		
	CON	CBA	ECON	ECBA		CBA	ETEC	Interaction
Duodenum								
AKP, U/g prot	5.00	5.94	5.17	5.33	0.36	0.47	0.77	0.61
Lactase, U/mg prot	79.67	79.22	75.05	81.60	2.39	0.54	0.82	0.48
Sucrase, U/mg prot	71.50	81.49	72.63	82.06	2.90	0.11	0.89	0.96
Maltase, U/mg prot	95.75^b^	129.14^ab^	100.00^b^	142.03^a^	6.70	<0.01	0.48	0.72
Jejunum								
AKP, U/g prot	8.35	8.74	9.52	6.29	0.52	0.16	0.52	0.08
Lactase, U/mg prot	82.88^a^	78.53^ab^	59.01^b^	76.09^ab^	3.45	0.33	0.05	0.11
Sucrase, U/mg prot	102.37^a^	96.35^a^	78.75^b^	100.72^a^	3.17	0.17	0.10	0.02
Maltase, U/mg prot	209.48	192.38	171.11	190.50	6.90	0.93	0.15	0.19
Ileum								
AKP, U/g prot	3.72^b^	6.02^a^	2.59^b^	2.56^b^	0.41	0.07	<0.01	0.06
Lactase, U/mg prot	93.60^b^	108.39^ab^	92.01^b^	120.03^a^	4.50	0.02	0.56	0.44
Sucrase, U/mg prot	70.88^ab^	74.30^ab^	56.75^b^	87.42^a^	3.17	<0.01	0.93	0.02
Maltase, U/mg prot	186.00^ab^	201.96^a^	152.89^b^	203.15^a^	7.16	0.02	0.23	0.20

AKP, alkaline phosphatase.

Mean and total SEM are list in Separate columns, *n* = 8.

a, b, c mean values within a row with unlike superscript letters were significantly different, *P* < 0.05.

CON, pigs were fed with a basal diet; CBA, pigs were fed with a CBA containing diet, 3 g/kg; ECON, pigs were fed with a basal diet and challenged by ETEC; ECBA, pigs were fed with a CBA containing diet and challenged by ETEC.

### 3.4 Effect of CBA supplementation on expression of intestinal barrier and nutrient absorption genes in weaned pigs after ETEC challenge

**Figure 3:**
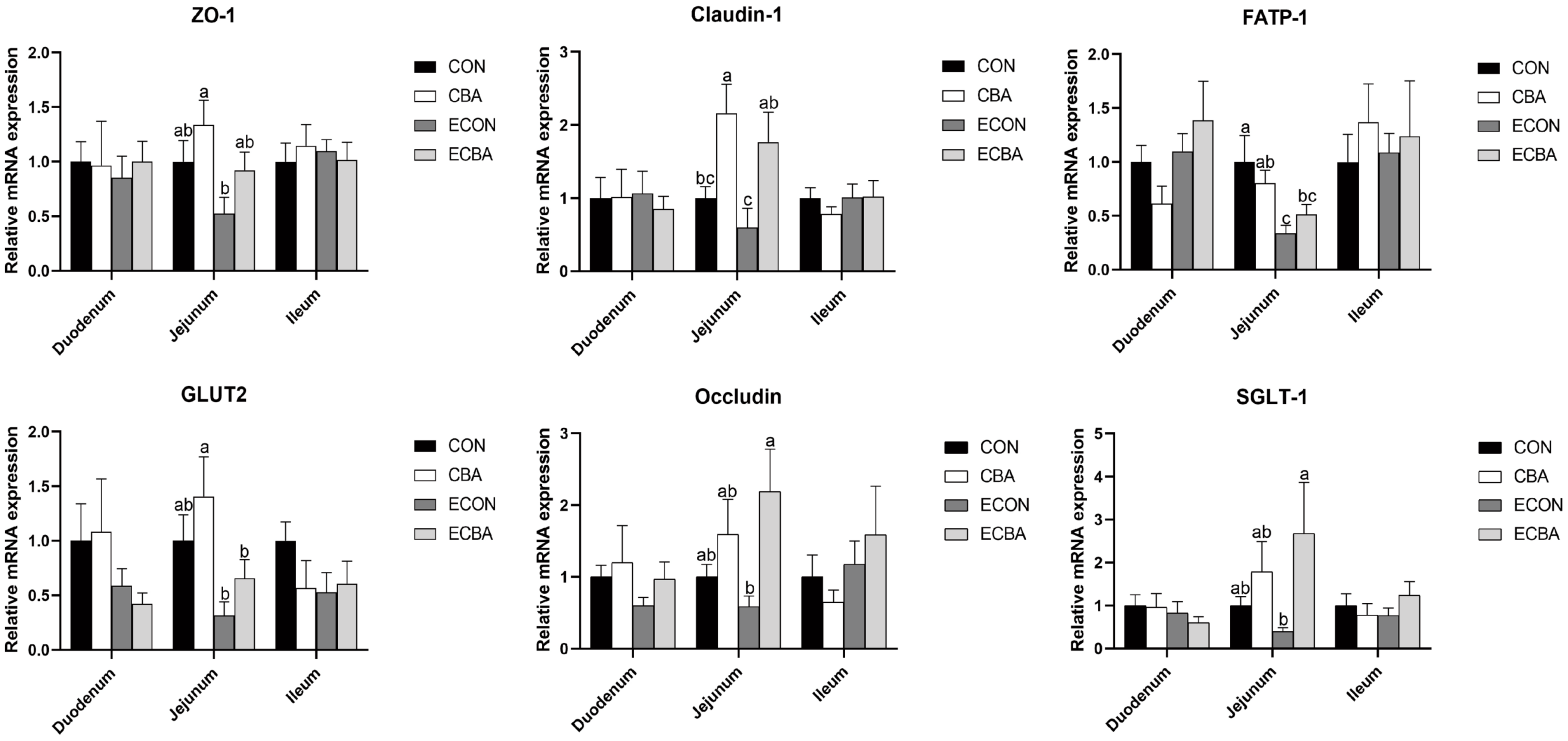
Effect of CBA supplementation on mucosal gene expressions in weaned pigs after ETEC challenge. ZO-1, zonula occludens-1; FATP, fatty acid transport proteins; GLUT-2, glucose transporter-2; CAT-1, cationic amino acid transporter-1; a, b, c mean values within a row with unlike superscript letters were significantly different (*p* < 0.05). CON, pigs were fed with a basal diet; CBA, pigs were fed with a CBA containing diet, 3 g/kg; ECON, pigs were fed with a basal diet and challenged by ETEC; ECBA, pigs were fed with a CBA containing diet and challenged by ETEC.

CBA supplementation tended to increase the expression levels of zonula occludens-1 (ZO-1) and glucose transporter-2 (GLUT2) genes in the jejunal mucosa of the non-challenged pigs (*p* < 0.1; Figure 3). CBA supplementation tended to increase the expression levels of the ZO-1 and fatty acid transport protein 1 (FATP-1) genes in the jejunal mucosa of the ETEC-challenged group (*p* < 0.1). Moreover, the ETEC challenge significantly reduced the expression levels of FATP-1 in the jejunal mucosa of piglets (*p* < 0.05). The expression levels of the Occludin and sodium/glucose cotransporter-1 (SGLT-1) genes were significantly higher in the ECBA group when compared with the ECON group (*p* < 0.05). CBA supplementation significantly elevated the expression levels of claudin-1 in the jejunal epithelium of the non-challenged as well as the ETEC-challenged pigs (*p* < 0.05).

### 3.5 Effect of CBA supplementation on intestinal microbial populations and metabolites in weaned pigs after ETEC challenge

CBA supplementation showed a 6.06% abundance of Bacillus elevation in the cecum of the non-challenged pigs (*p* < 0.05; Table 8) and a 1.89% total bacterial content increase in the cecum (*p* < 0.05). CBA supplementation enhanced the abundance of acetic (26.63%), propionic (34.84%), and butyric acid (37.59%) levels in the cecum of the non-challenged pigs (*p* < 0.05). ETEC challenge increased colonic *E. coli* (19.00%) and total bacterial counts (2.42%) compared to the CON group, but CBA supplementation tended to decrease colonic *E. coli* and total bacterial counts. ETEC challenge showed a 31.33% propionic acid decrease in the colon compared to the CON group, but CBA supplementation tended to increase colonic propionic acid.

**Table 8:** Effect of CBA supplementation on caecal microbial populations and its products in weaned pigs after ETEC challenge.

ITEM	Treatments				SEM	*p*-value		
	CON	CBA	ECON	ECBA		CBA	ETEC	Interaction
Cecum, microbial populations, lg (copies/g)								
Total bacteria	11.09^b^	11.30^a^	10.98^b^	10.99^b^	0.03	0.04	<0.01	0.05
*Lactobacillus*	8.59	8.71	8.77	8.46	0.10	0.65	0.88	0.31
*Escherichia coli*	9.11	9.60	10.31	9.56	0.20	0.74	0.15	0.12
*Bifidobacterium*	7.03	7.28	6.83	6.53	0.22	0.95	0.30	0.54
*Bacillus*	9.24^b^	9.80^a^	9.44^ab^	9.38^b^	0.07	0.08	0.42	0.03
*Colon*, microbial populations, lg (copies/g)								
Total bacteria	11.18^b^	11.42^a^	11.45^a^	11.27^ab^	0.04	0.66	0.43	<0.01
*Lactobacillus*	8.70	8.88	8.49	8.82	0.11	0.27	0.56	0.74
*Escherichia coli*	8.84^b^	9.31^ab^	10.52^a^	10.10^ab^	0.24	0.95	<0.01	0.31
*Bifidobacterium*	7.48	6.94	7.25	6.95	0.22	0.37	0.82	0.79
*Bacillus*	9.59	9.42	9.41	9.56	0.05	0.92	0.84	0.16
Cecum, VFA, mg/g								
Acetic acid	4.13^b^	5.23^a^	3.97^b^	3.37^b^	0.21	0.47	<0.01	0.02
Propanoic acid	2.44^b^	3.29^a^	2.25^b^	1.98^b^	0.14	0.19	<0.01	0.02
Butyric acid	1.41^b^	1.94^a^	1.43^b^	1.02^b^	0.10	0.72	0.01	<0.01
*Colon*, VFA, mg/g								
Acetic acid	4.72	5.10	3.93	4.55	0.26	0.35	0.21	0.82
Propanoic acid	3.00^a^	3.28^a^	2.06^b^	2.93^ab^	0.17	0.08	0.05	0.33
Butyric acid	1.08	1.55	1.22	1.10	0.10	0.40	0.46	0.16

VFA, volatile fatty acids.

Mean and total SEM are list in Separate columns, *n* = 8.

a, b, c mean values within a row with unlike superscript letters were significantly different, *P* < 0.05.

CON, pigs were fed with a basal diet; CBA, pigs were fed with a CBA containing diet, 3 g/kg; ECON, pigs were fed with a basal diet and challenged by ETEC; ECBA, pigs were fed with a CBA containing diet and challenged by ETEC.

## 4 Discussion

BA is an antimicrobial agent with a broad spectrum of activity against pathogenic bacteria (27). Previous studies have found that BA had a positive effect on growth performance in piglets, which was related to antibacterial activity, improving nutrient digestion and absorption (28), maintaining the gastrointestinal tract environment (29), and promoting the production and activation of digestive enzymes (30). However, BA is usually added to animal diets in a free form, and its rapid absorption limits its ability to exert its potential antimicrobial and immunomodulatory effects outside the stomach and proximal small intestine (31). Here, CBA was supplemented to the diets of weaned piglets to enable it to act throughout the gastrointestinal tract. In this study, we found that CBA not only increased the ADG of the ETEC-challenged pigs, but also increased the digestibility of DM, CP, Ash, and GE in weaned piglets before the ETEC challenge, which is consistent with previous studies (32). Both results show a beneficial effect of CBA supplementation on the growth performance of the weaned pigs.

Changes in blood leukocytes are important indicators of immune system status (33). Basophils are effector cells of innate immunity, as the primary purveyor of blood histamine, and play a central role in the pathogenesis of protective immunity (34). CBA supplementation significantly increased the percentage of basophils of the ETEC-challenged pigs, indicating an immune enhancement in the pigs after ETEC challenge. Serum biochemistries are routine blood tests that are closely related to nutritional status, protein metabolism, and disease. The fluctuation of serum biochemical indexes can reflect the body’s metabolism and health (35).

The levels of serum albumin and urea are closely connected with protein digestion and absorption. In particular, serum urea is the main end product of protein metabolism in mammals (36). This study found that adding CBA to the diet increased serum albumin content while decreasing serum urea content. This suggests that protein utilization was improved in the CBA dietary treatments, which supports the previously mentioned improvement in ADG. Total cholesterol reflects the rate of lipid metabolism and liver health (37). In this study, CBA supplementation increased the content of serum total cholesterol of the non-challenged pigs, suggesting that CBA may affect lipid metabolism. Immunoglobulins, also known as antibodies, are a class of glycoproteins produced by plasma cells (38). They primarily exist in the serum and intestinal mucosa, providing cell protection against pathogenic viruses and microorganisms (39). As one of the constituents of the animal immune system, IgA is the second most abundant immunoglobulin in serum (40), IgG mainly kills bacteria in the serum, whereas IgM aids in preventing bacterial and fungal infections for it plays an important role in promoting mucosal tolerance and creating a healthy microecological environment within the intestine (41). CBA significantly elevated the serum IgA and IgG concentrations and demonstrated a tendency to increase IgM concentration of the ETEC-challenged pigs, indicating enhanced immunity with CBA supplementation.

The small intestine plays a pivotal role in digesting, absorbing, and transporting nutrients, with its villi being indispensable for these functions. Hence, evaluation of intestinal digestion was carried out by measuring the villous height to crypt depth ratio (42). The higher villi favor a greater absorption of nutrients by the digestive system. A lower CD value indicates a greater population of mature epithelial cells, while a higher VH:CD ratio suggests a more favorable mucosal structure with a larger digestive and absorptive capacity (43). However, the intestinal morphology can be impaired after ETEC infection (44). Previous research found that pH value would affect cell growth, and the cell division would be improved as the acidity increased (45). Other research found that the reduction of intestinal villus height and increase of crypt depth of piglets was related to the decrease of sucrase and lactase activities in the striated border (46). CBA supplementation significantly increased the activities of maltase in the duodenum and sucrose in the jejunum. CBA supplementation also enhanced the ileal activity of lactase, sucrose and maltase of the ETEC-challenged pigs, and of alkaline phosphatase of the non-challenged pigs, which suggested that CBA supplementation can alleviate intestinal stress in piglets by reducing the pH value of the intestine, increasing the activity of digestive enzymes in the small intestine, improving intestinal morphology, and maintaining the health of the intestinal mucosa after weaning. Tight junctions play a crucial role in intestinal barrier integrity and protect pathogenic bacteria colonization and macromolecular transmission from paracellular diffusion (47), composed of transmembrane barrier proteins (e.g., claudins and occludin), cytoplasmic scaffold proteins (e.g., ZO family), and adhesion molecules. Claudins are integral membrane proteins that are primarily found at tight junctions. They play a critical role in apical cell-to-cell adhesion, maintenance of epithelial polarity, and formation of impermeable barriers between epithelial cells (48). In this study, we found that CBA supplementation significantly elevated the expression levels of claudin-1 in the jejunal epithelium of the non-challenged as well as the ETEC-challenged pigs, and significantly elevated the expression levels of Occludin and SGLT-1 in the jejunal epithelium of the ETEC-challenged pigs. CBA supplementation also tended to increase the expression levels of ZO-1. Moreover, CBA also elevated the expression levels of GLUT2 and FATP-1 in the jejunal epithelium. In pig intestine, SGLT1 is the major route for absorption of dietary glucose from the lumen of the intestine into enterocytes (49). FATP-1 plays an important role in the regulation of fatty acid metabolism and lipid accumulation (50), and GLUT-2 is one of the major transporters for glucose absorption. These results suggest that CBA administration can improve nonspecific barrier mechanisms and epithelial in the gut.

Intestinal microbial populations are associated with nutrient digestion, absorption, and gut health (51). Gut microbes play a crucial role in maintaining gut health. The balance of beneficial bacteria such as *Lactobacillus*, *Bifidobacterium*, and *Bacillus*, and the reduction of harmful bacteria like *Escherichia coli*, are associated with intestinal morphology and can help prevent diarrhea (52). In this study, we found that CBA supplementation significantly elevated the abundance of beneficial bacteria, *Bacillus* in the cecum of the non-challenged pigs, and had a tendency to decrease colonic *Escherichia coli* counts. The beneficial bacteria suppress the colonization of *Escherichia coli* by blocking the sites of adhesion and producing acidic metabolites (17), which might be the partial explanation for why CBA could regulate the intestinal microbiota. The change in gut microbial composition leads to the variation in microbial metabolites. As microbial metabolites, VFAs have a function that is similar to inhibiting bacteria (53), and they are not only positive in providing energy for intestinal epithelial cells but promote the formation of cells by stabilizing DNA and repairing damage, thus promoting the proliferation of epithelial cells (54). Furthermore, VFA can increase the acidity of intercellular substances in harmful bacteria to inhibit bacteria, destroy the balance of osmotic pressure in harmful bacteria, and thus play a role in regulating microflora (53). CBA supplementation excellently enhanced the abundance of acetic, propionic, and butyric acid levels in the cecum of the non-challenged pigs, and had a tendency to decrease colonic *Escherichia coli* counts of the ETEC-challenged pigs. CBA not only modulates the intestinal microflora but also increases the concentration of VFA. This provides new explanations for the positive effects of CBA on gut health.

In the present study, we found that CBA has beneficial effects on intestinal morphology, expression of tight junction genes and nutrient absorption genes, probiotic colonization and VFA production (55). Previous studies have shown that probiotics such as *Bacillus* have a protective effect on the intestinal barrier. Therefore, our hypothesis that CBA could improve intestinal function by stimulating the growth of beneficial bacteria and suppressing the growth of potential pathogenic bacteria, increasing microbial fermentation and breakdown of complex carbohydrates, and increasing VFA production was correct.

## Data Availability

The raw data supporting the conclusions of this article will be made available by the authors, without undue reservation.
