# Supplementary material for: Effect of coated-benzoic acid on growth performance, immunity, and intestinal functions in weaned pigs challenged by enterotoxigenic Escherichia coli
Source: Front Vet Sci. 2024 Sep 16;11:1430696. doi: 10.3389/fvets.2024.1430696 (PMC11439879; doi:10.3389/fvets.2024.1430696)
Supplement: Supplementary file 1 [file Table_1.DOCX]

Table S1. Sequences of primers for genes and intestinal bacteria

| Gene | Primer sequence (5’ to 3’) | Annealing temperature, ℃ | Product size, bp |
| --- | --- | --- | --- |
| β-Actin | F: TGGAACGGTGAAGGTGACAGC | 60 | 177 |
|  | R: GCTTTTGGGAAGGCAGGGACT |  |  |
| GLUT-2 | F: TGGAATCAGCCAACCTGTTT | 60 | 146 |
|  | R: ACAAGTCCCACCGACATGA |  |  |
| SGLT-1 | F: AGCTACCTCAAGATGCTGCC | 60 | 192 |
|  | R: ATCATCTGGAAACAGCACGC |  |  |
| FATP-1 | F: GGAGTAGAGGGCAAAGCAGG | 60 | 208 |
|  | R: AGGTCTGGCGTGGGTCAAAG |  |  |
| ZO-1 | F: CAGCCCCCGTACATGGAGA | 60 | 114 |
|  | R: GCGCAGACGGTGTTCATAGTT |  |  |
| Occludin | F: CTACTCGTCCAACGGGAAAG | 60 | 158 |
|  | R: ACGCCTCCAAGTTACCACTG |  |  |
| Claudin-1 | F: TCTTAGTTGCCACAGCATGG | 60 | 135 |
|  | R: CCAGTGAAGAGAGCCTGACC |  |  |
| Total bacteria | F: ACTCCTACGGGAGGCAGCAG | 60 | 200 |
|  | R: ATTACCGCGGCTGCTGG |  |  |
| *Lactobacillus* | F: GAGGCAGCAGTAGGGAATCTTC | 60 | 126 |
|  | R: CAACAGTTACTCTGACACCCGTTCTTC |  |  |
|  | P: AAGAAGGGTTTCGGCTCGTAAAACTCTGTT |  |  |
| *Escherichia coli* | F: CATGCCGCGTGTATGAAGAA | 60 | 96 |
|  | R: CGGGTAACGTCAATGAGCAAA |  |  |
|  | P: AGGTATTAACTTTACTCCCTTCCTC |  |  |
| *Bifidobacterium* | F: CGCGTCCGGTGTGAAAG | 60 | 121 |
|  | R: CTTCCCGATATCTACACATTCCA |  |  |
|  | P: ATTCCACCGTTACACCGGGAA |  |  |
| *Bacillus* | F: GCAACGAGCGCAACCCTTGA | 60 | 92 |
|  | R: TCATCCCCACCTTCCTCCGGT |  |  |
|  | P: CGGTTTGTCACCGGCAGTCACCT |  |  |

*SGLT-1*, sodium/glucose cotransporter 1; *GLUT-2*, glucose transporter 2; *FATP-1*, fatty acid transport protein 1; *ZO-1*, zonula occludens 1.
